# Clinical features, therapeutic interventions and long-term aspects of hemolytic-uremic syndrome in Norwegian children: a nationwide retrospective study from 1999–2008

**DOI:** 10.1186/s12879-016-1627-7

**Published:** 2016-06-13

**Authors:** Gaute Reier Jenssen, Line Vold, Eirik Hovland, Hans-Jacob Bangstad, Karin Nygård, Anna Bjerre

**Affiliations:** Department of Infectious Disease Epidemiology, Norwegian Institute of Public Health (Nasjonalt Folkehelseinstitutt), Postboks 4404, Nydalen, NO 0403 Oslo, Norway; Institute of Clinical Medicine, Faculty of Medicine, University of Oslo, Oslo, Norway; Department of Pediatrics, Oslo University Hospital, Oslo, Norway

**Keywords:** Enterohaemorrhagic *E. coli* - EHEC, Epidemiology, Haemolytic uraemic syndrome, Shiga toxin producing *E. coli – STEC*, clinical outcome, aHUS, SP-HUS

## Abstract

**Background:**

Hemolytic-uremic syndrome (HUS) is a clinical triad of microangiopathic hemolytic anemia, impaired renal function and thrombocytopenia, primarily affecting pre-school-aged children. HUS can be classified into diarrhea-associated HUS (D^+^HUS), usually caused by Shiga toxin-producing *Escherichia coli* (STEC), and non-diarrhea-associated HUS (D^−^HUS), both with potentially serious acute and long-term complications. Few data exists on the clinical features and long-term outcome of HUS in Norway. The aim of this paper was to describe these aspects of HUS in children over a 10-year period.

**Methods:**

We retrospectively collected data on clinical features, therapeutic interventions and long-term aspects directly from medical records of all identified HUS cases <16 years of age admitted to Norwegian pediatric departments from 1999 to 2008. Cases of D^+^HUS and D^−^HUS are described separately, but no comparative analyses were possible due to small numbers. Descriptive statistics are presented in proportions and median values with ranges, and/or summarized in text.

**Results:**

Forty seven HUS cases were identified; 38 D^+^HUS and nine D^−^HUS. Renal complications were common; in the D^+^HUS and D^−^HUS group, 29/38 and 5/9 developed oligoanuria, 22/38 and 3/9 needed dialysis, with hemodialysis used most often in both groups, and plasma infusion(s) were utilized in 6/38 and 4/9 patients, respectively. Of extra-renal complications, neurological complications occurred in 9/38 and 2/9, serious gastrointestinal complications in 6/38 and 1/9, respiratory complications in 10/38 and 2/9, and sepsis in 11/38 and 3/9 cases, respectively. Cardiac complications were seen in two D^+^HUS cases. In patients where data on follow up ≥1 year after admittance were available, 8/21 and 4/7 had persistent proteinuria and 5/19 and 4/5 had persistent hypertension in the D^+^HUS and D^−^HUS group, respectively. Two D^+^HUS and one D^−^HUS patient were diagnosed with chronic kidney disease and one D^+^HUS patient required a renal transplantation. Two D^+^HUS patients died in the acute phase (death rate; 5 %).

**Conclusions:**

The HUS cases had a high rate of complications and sequelae, including renal, CNS-related, cardiac, respiratory, serious gastrointestinal complications and sepsis, consistent with other studies. This underlines the importance of attention to extra-renal manifestations in the acute phase and in renal long-term follow-up of HUS patients.

## Background

Hemolytic-uremic syndrome (HUS) is a clinical condition characterized by the triad of impaired renal function, non-immune hemolytic anemia and thrombocytopenia, and is considered one of the most common causes of acute kidney injury (AKI) in children in Europe and the Western world [1–3]. HUS mainly affects children of pre-school age [4]. In Norway, HUS is the second most common cause of AKI in children, and has an estimated average annual incidence rate of 0.5 cases per 100,000 children [5, 6]. This is lower than in most European countries [7–9].

A common classification of HUS is by clinical presentation; associated with prodromal diarrhea (D^+^HUS) or not (D^−^HUS). Around 90 % of HUS cases in children are D^+^HUS [7, 10]. In the Western world, most cases of D^+^HUS are caused by infection with Shiga toxin producing *Escherichia coli* (STEC-HUS) [4]. According to this classification, D^−^HUS mainly consists of HUS caused by *Streptococcus pneumoniae* infection (SP-HUS) and HUS associated with familiar or sporadic genetic disorders of complement regulation (atypical HUS; aHUS) [11]. This classification has some limitations. STEC-HUS is generally considered D^+^HUS, although some cases may present without diarrhea [4]. Some of the aHUS cases may present with diarrhea, but are eventually classified as D^−^HUS. Therefore, other classifications define HUS based on both clinical associations and causal factors [12, 13]. It has been suggested that some STEC-HUS cases, especially those with more severe outcome, are genetically predisposed aHUS cases triggered by an STEC infection [14].

D^+^HUS patients usually present with signs of enteropathic infection; diarrhea, often bloody and/or watery, abdominal tenderness and more rarely low grade fever [15]. Renal affection with decreasing diuresis and subsequent oliguria and/or anuria usually follows in the estimated 10–15 % who develops HUS, although temporary renal impairment can be seen due to dehydration in STEC infections without HUS. Symptoms and complications from extrarenal involvement may occur in the acute phase; most often from the central nervous system (CNS), but also of respiratory, cardiac and gastrointestinal nature [16–18]. D^−^HUS may present with various and prolonged atypical symptoms [11].

The clinical features of HUS are a consequence of microvascular lesions termed thrombotic microangiopathy (TMA). TMA mainly affects arterioles and capillaries of the kidneys and the CNS and results in impaired blood vessel flow with subsequent ischemic damage in the affected organs [4, 19].

Long-term sequelae of HUS are predominantly renal with reduced glomerular filtration rate, hypertension and/or prolonged proteinuria [4]. In a large meta-analysis, it was estimated that renal sequelae without end stage renal disease (ESRD) occurred in around 25 % and an outcome of ESRD in 3 % of D^+^HUS cases [20]. The death rate is considered 3–5 %, but varies between studies [20, 21]. D^+^HUS and D^−^HUS are associated with similar short and long-term complications, but clinical signs of kidney dysfunction are considered more frequent in the latter. The death rate is higher in SP-HUS than D^+^HUS, especially in the acute phase, although the long-term renal prognosis for this group is generally favorable [11, 22].

Treatment of HUS has until recently been supportive: fluid therapy, dialysis, plasmapheresis/plasma infusion and treatment of complications [4, 23, 24]. The emergence of eculizumab, a monoclonal C5 antibody, has now provided a proven effective treatment of genetic aHUS [25]. Eculizumab has also shown potential in the treatment of D^+^HUS, and further studies are currently ongoing [26].

Knowledge on HUS in Norway has been limited. The first national outbreak of STEC-HUS in Norway, in which one child died, occurred in 2006 [27]. This outbreak led to the notification criteria of HUS being changed from STEC-HUS to all D^+^HUS and brought HUS to public attention [28]. We recently published national data focusing on the epidemiological and surveillance aspects of HUS in children in Norway [5]. There, we concluded that the incidence of HUS was low compared to most European countries, but higher than previously assumed. STEC-HUS is the second most common cause of acute kidney injury in children in Norway [6], but national data on sequelae and outcomes has not been presented before. The primary aim of the current study is to describe the clinical features, therapeutic interventions and long-term aspects of the cases of D^+^HUS and D^−^HUS included in the epidemiological study.

## Methods

### Design

We performed a retrospective, descriptive study of data collected directly from relevant patient medical records.

### Data collection

Potentially relevant cases were identified through medical record searches for pediatric patients < 16 years of age initially admitted to a Norwegian hospital from the 1st of January, 1999, to the 31^st^ of December, 2008, with ICD-10 codes D59.3 (HUS), N17 (AKI) and/or N00/N01/N05 (acute nephritic syndrome/rapidly progressive nephritic syndrome/unspecified nephritic syndrome). The non-HUS ICD-10 codes were included in the search and evaluated on site to identify potentially misdiagnosed cases of HUS. Cases matching the case definitions seen below were included. Cases that had been partially treated in Norwegian pediatric departments, but had initially been admitted for HUS outside of Norway, were excluded.

All Norwegian hospitals with pediatric capacity were contacted prior to the data collection to identify relevant cases. Data were collected from relevant patient medical records from 24 pediatric departments of Norwegian hospitals; directly from 18 hospitals. Data from the remaining six hospitals were collected indirectly as they confirmed in advance having transferred all relevant patients to one of the former 18 hospitals. Medical record data missing from the six hospitals were obtained by mail. Data were collected on site by two project co-workers (medical students/authors Jenssen and Hovland), with two pediatric nephrologists (authors Bangstad and Bjerre) available for phone consultations in unclear and/or difficult cases. The coordinating center of the study was the Norwegian Institute of Public Health.

Forms made with EpiData (www.epidata.dk) were used to register data. The forms were designed through a pilot project, in which we examined the availability of relevant variables in standard medical records. The registered data were stored and encrypted according to the information security standards of the Norwegian Institute of Public Health.

### Case definitions

A hemolytic-uremic syndrome (HUS) case was defined as:

- a case clinically compatible with all the following laboratory findings of
  - ○ thrombocytopenia (<150 × 10^9/L), AND
  - ○ anemia (Hgb < 10.5 g/dL) of hemolytic origin, with elevated serum lactate dehydrogenase (LD) (>500 U/L), AND
  - ○ acutely reduced renal function (serum creatinine >35 μmol/L for patients < 1 years of age, > 80 μmol/L for patients 1–15 years of age), AND EITHER
    - ▪ Reported presence of fragmented red blood cells (schiztocytes) on peripheral blood smear; a sign of microangiopathic changes consistent with hemolysis and an important part of HUS pathophysiology [1], or
    - ▪ if peripheral blood smear was missing in the journal; probable clinical HUS confirmed by consulting a clinician with expertise in pediatric nephrology

A diarrhea-associated HUS (D^+^HUS) case was defined as a HUS case with either:

- a clinical presentation of prodromal diarrhea, without verifiable causative etiology (probable STEC-HUS), OR
- STEC-HUS, defined as a HUS case with laboratory-verified STEC-infection

A D^−^HUS case was defined as any non-diarrhea-associated HUS or HUS of verified non-STEC causality.

### Variables collected

The following clinical variables were collected: time from first symptom to admittance; age at admittance; duration of initial hospitalization; duration of total time hospitalized; presence of prodromal diarrhea; presence of prodromal bloody diarrhea; presence of hypertension at admittance; development of oligoanuria, hypertension and/or proteinuria in the acute phase; extra-renal complications; death in the acute phase; laboratory values at admission and minimal/maximal value (hemoglobin, creatinine, LD, platelet count, CRP, white blood cell count, sodium).

The following therapeutic intervention variables were collected: use of dialysis, type of dialysis used, duration of dialysis; use of plasmapheresis, red blood cell transfusions, platelet transfusions, plasma infusions/exchange, antibiotics (any indication); renal transplantation performed; use of other therapeutic modalities.

The following long-term/outcome variables were collected: presence of hypertension and/or proteinuria at first follow-up and at follow up 1 year or more following initial admission; presence of renal sequelae/long-term complications; estimated glomerular filtration rate and/or creatinine value at first follow-up and at follow up 1 year or more following initial admission; death at follow up.

### Statistical analysis

Descriptive statistics were calculated using Microsoft Excel and are presented as proportions and median values with ranges. Variables are presented in tables and/or in text. Estimated glomerular filtration rate (eGFR) was estimated retrospectively using the height-independent Pottel eGFR equation [29]. Descriptive analyses for the D^+^HUS and D^−^HUS group were done separately, but no comparative analyses were done between the two groups due to the small number of D^−^HUS cases.

## Results

### Patients

Forty-seven HUS patients, (16 boys and 31 girls; median age 2 years, range 5 months to 15 years) were identified from 1999 up to and including 2008. 38 (81 %) were D^+^HUS cases, 23 (61 %) of which had confirmed STEC infection. Nine (19 %) were D^−^HUS cases; two SP-HUS, three of verified genetic origin, one specified as *Campylobacter*-related, and three non-diarrhea-associated cases with unknown etiology. The genetic HUS cases all had CD46-mutations; one had an additional C3-mutation, another had antibodies to Factor H [5].

All but one of the D^+^HUS cases presented with diarrhea and 27 (71 %) of these had bloody stools. The D^+^HUS case presenting without diarrhea had confirmed STEC infection. Two (22 %) of the nine patients with D^−^HUS presented with diarrhea, both had bloody stools. One initially presented with non-bloody diarrhea, clinical HUS and mild infection parameters. Bloody diarrhea was only noted after transfer to a larger hospital. There the patient developed bacteremia from a central venous catheter-related *Staphylococcus aureus* infection and had *Enterococcus faecalis* identified in a urine sample. This patient later had HUS relapses and was shown to carry both a CD46 and a C3-mutation. The other D^−^HUS patient was the above mentioned were only *Campylobacter jejuni* was isolated in stool samples and specified as cause in the medical record. Both were defined as D^−^HUS cases due to the etiological cause.

For the D^+^HUS and D^−^HUS group, respectively, median time from first registered symptom to admittance was 6 and 5 days, initial hospitalization lasted a median 15 and 16 days, whereas 29/38 (76 %) and 5/9 (56 %) cases developed oligoanuria at some point during initial admission (Table 1). Two of the D^+^HUS patients died, both in the acute phase, with a death rate of 5 %. None of the D^−^HUS cases had died at the point of data assessment.

**Table 1:** Clinical features of HUS in children in Norway, 1999-2008

Clinical feature	Diarrhoea-associated HUS (*N* = 38)	Non-diarrhea-associated HUS (*N* = 9)
Time first symptom to admittance (median, days)	6 (4–9)	5 (2–10)
Age at admittance (median, months/years)^a^	31 (range; 5 months–15 years)^a^	18 (range; 7 months–6 years)^a^
Duration of initial hospitalization (median, days)	15 (11–24)	16 (8–42)
Duration of total time hospitalized^b^ (median, days)	18 (12–24)	16 (8–53)
Prodromal diarrhea (*n*, %)	37 (97 %)	2 (22 %)
Prodromal bloody diarrhea (*n*, %)	27 (71 %)	2 (22 %)
Hypertension at admittance (*n*, %)	4 (24 %) (*N* = 17)	2 (33 %) (*N* = 6)
Hypertension registered during admittance (*n*, %)	30 (83 %) (*N* = 36)	8 (100 %) (*N* = 8)
Oligoanuria (*n*, %)	29 (76 %)	5 (56 %)
Death acute phase (*n*, %)	2 (5 %)	0 (0 %)
*Non-renal complications*		
Neurological complications (*n*, %)	9 (24 %)	2 (22 %)
Cardiac complications (*n*, %)	2 (5 %)	0 (0 %)
Respiratory complications (*n*, %)	10 (26 %)	2 (22 %)
Gastrointestinal complications (*n*, %)	5 (13 %)	1 (11 %)
Sepsis (*n*, %)	11 (29 %)	3 (33 %)
*Renal outcome*		
Proteinuria at first follow-up (*n*, %)	16 (50 %) (*N* = 32)	7 (78 %)
Proteinuria ≥ 1 year after initial admission (*n*, %)	8 (38 %) (*N* = 21)	4 (57 %) (*N* = 7)
Hypertension at first follow-up (*n*, %)	10 (31 %) (*N* = 32)	5 (56 %)
Hypertension ≥ 1 year after initial admission (*n*, %)	5 (26 %) (*N* = 19)	4 (80 %) (*N* = 5)
Chronic kidney disease (*n*, %)	2 (5 %)	1 (11 %)
End-stage renal disease (ESRD)	1 (3 %)	0 (0 %)

Results are presented as number of cases, *n* (%) and median (interquartile range). If data on the feature was not available in all medical records, the number of cases where available is presented (*N* = number of cases where available). *HUS* hemolytic uremic syndrome

^a^Range; smallest and highest value for illustrational purposes

^b^Time hospitalized including all readmissions for complications and extensive (not regular) follow-up

Few patients had registered blood pressure at admittance. In the D^+^HUS and D^−^HUS group, respectively, 4/17 (24 %) and 2/6 (33 %) cases were hypertensive. However, 30/36 (83 %) D^+^HUS cases and all eight D^−^HUS cases where information on blood pressure was available had registered hypertension at some point during initial admission (Table 1).

### Non-renal clinical features of D^+^HUS patients (Table 1)

Different neurological complications were seen in nine (24 %) of 38 cases in the D^+^HUS group, and manifested as follows; two patients with mild brain infarctions; one with brain microinfarctions; two patients developed brain edema; one developed brain tamponade; one had clinical meningitis; one developed intracranial hematoma following a procedure; one suffered anoxic brain damage, with brain atrophy and epilepsy. Four of the patients had seizures in the acute phase, including two without further neurological complications. In one patient, brain scanning showed lowered white matter echogenicity. Finally, one patient showed signs of CNS affection, manifested as an inability to remember certain words.

Cardiac complications were seen in two (5 %) patients; one had multiple myocardial infarctions and cardiac arrest with successful resuscitation, the other developed pericardial fluid effusion following an episode of sepsis.

Respiratory complications were described in ten (26 %) cases, with the need of ventilation therapy described in nine patients. One patient had pneumothorax. Three had hydrothorax, one of which did not receive ventilation therapy. One developed pulmonary collapse and chronic respiratory failure. The remaining patients needed ventilation therapy in the process related to other complications listed here, including sepsis and neurological events.

Five (13 %) patients had serious gastrointestinal complications. Two patients developed colonic necrosis with perforation, peritonitis and sepsis, requiring left hemicolectomy and subtotal colectomy, respectively. Two patients experienced gall stone problems, one of them requiring cholecystostomy. Other gastrointestinal complications included one patient with rectal prolapse and two with intestinal invagination. One patient in the D^+^HUS group also had pancreatic complications, with the developement of diabetes mellitus.

There were eleven (29 %) cases complicated by sepsis. *Staphylococcus aureus* was specified as causative agent in two, *Staphylococcus epidermidis* in another two. One was caused by streptococcal throat infection, one by *Acinetobacter baumannii* and one had urosepsis but the agent was not specified. In the remaining cases, we were unable to identify the causative agent; two cases were complications of a perforated intestine and STEC infection proven in four without conclusive evidence of causing sepsis. Two patients developed septic shock; in both, STEC (serotype O87 and O103, respectively) was the only agent identified.

### Non-renal clinical features of D^−^HUS patients (Table 1)

The most severe symptoms were in the two SP-HUS patients with septicemia, neurological and respiratory complications. Complications included pneumococcal-induced septic meningitis with acute respiratory failure, development of brain atrophy, hemiplegia with spastic convulsions and epileptic activity, neuronal hearing loss and retinopathy, seizures related to pneumococcal septic pneumonia with pleural empyema and acute respiratory failure. Both patients needed ventilator therapy.

The third case of sepsis in the D^−^HUS group was of *Staphylococcus aureus* origin, after complications with a central venous catheter.

One D^−^HUS patient developed gall-stone problems during admission, eventually needing endoscopic retrograde cholangiopancreatography-guided extraction.

### Long term sequelae (Table 1)

Follow up data 1 year or more after initial admission were available in more than half the cases. With the exception of one case from 2008, all D^+^HUS medical records were assessed at least 1 year after being diagnosed with HUS. The D^−^HUS medical records were assessed a minimum of 2 years following primary admission.

At the first follow up after being released from hospital, presence of persistent proteinuria was seen in 16/32 (50 %) cases in the D^+^HUS group and 7/9 (78 %) cases in the D^−^HUS group. At follow up at 1 year or more following initial admission, presence of persistent proteinuria was seen in 8/21 (38 %) cases and 4/7 (57 %) cases, respectively. Persistent hypertension was seen in 10/32 (31 %) cases in the D^+^HUS group and 5/9 (56 %) cases in the D^−^HUS group at the first follow up, and in 5/19 (26 %) and 4/5 (80 %) cases at follow up 1 year or more following initial admission, respectively.

Within the time frame from initial admission to last follow up and/or registered follow up assessed in the data collection, two (5 %) of the 36 D^+^HUS patients that survived the acute phase and one (11 %) of the D^−^HUS patients had been diagnosed with chronic kidney disease, and one had developed ESRD requiring renal transplantation.

### Therapeutic interventions (Table 2)

**Table 2:** Therapeutic interventions in HUS in children in Norway, 1999–2008

Therapeutical interventions	Diarrhoea-associated HUS (*N* = 38)	Non-diarrhea-associated HUS (*N* = 9)
Dialysis – any type (*n*, %)	22 (58 %)	3 (33 %)
Type of dialysis (n) − Peritoneal (*n*, %) − Hemodialysis (*n*, %) − Both (*n*, %)	(*N* = 22) 6 (27 %) 13 (59 %) 3 (14 %)	(*N* = 3) 1 (33 %) 2 (66 %) 0 (0 %)
Duration of dialysis (median, days)	8 (5–15) (*N* = 22^a^)	12 (7–13) (*N* = 3)
Plasmapheresis (*n*, %)	3 (8 %)	1 (11 %)
Red blood cell transfusion(s) (*n*, %)	34 (89 %)	9 (100 %)
Platelet transfusion(s) (*n*, %)	15 (39 %)	3 (33 %)
Plasma infusion(s) (*n*, %)	6 (16 %)	4 (44 %)
Antibiotics – any indication (*n*, %)	23 (61 %)	4 (44 %)
Ventilation therapy (*n*, %)	9 (24 %)	2 (22 %)
ERCP (*n*, %)	0 (0 %)	1 (11 %)
Cholecystostomy (*n*, %)	1 (3 %)	0 (0 %)
Renal transplantation (*n*, %)	1 (3 %)^b^	0 (0 %)

Results are presented as number of cases, *n* (%) and median (interquartile range). The values for type and duration of dialysis are estimated from those who received dialysis only, as specified (*N* = number of cases). *HUS* hemolytic uremic syndrome, *ERCP* endoscopic retrograde cholangiopancreatography

^a^Including the only patient that received dialysis after initial admission (for an additional 133 days until renal transplantation)

^b^12 months after initial admission

Table 2 presents the therapeutic interventions implemented for all HUS patients. In the D^+^HUS and D^−^HUS group, dialysis was performed in 22 (58 %) and three (33 %) cases, for a median duration of 8 and 12 days, and the most common modality was hemodialysis, utilized in 16/22 (73 %) and 2/3 (66 %) cases needing dialysis, respectively. Duration of dialysis was performed at primary admission only, with one exception; one patient never recovered kidney function and continued dialysis for an additional 133 days. Plasmapheresis was performed in three (8 %) and one (11 %), plasma infusions used in six (16 %) and four (44 %) and red blood cell transfusions used in 34 (89 %) and all of the cases in the D^+^HUS and D^−^HUS group, respectively. Antibiotics were given in 23 (61 %) of D^+^HUS cases and four (44 %) of D^−^HUS cases. However, time of administration was often unclear or not specified in the medical records. This also applied to indication for treatment, which included various conditions such as sepsis, catheter infection, pneumonia and urinary tract infection.

### Laboratory data (Table 3)

**Table 3:** Laboratory data in HUS in children in Norway, 1999–2008

Laboratory feature	Diarrhoea-associated HUS (*N* = 38)	Non-diarrhea-associated HUS (*N* = 9)
Hemoglobin at admission (median, g/dL)	11.1 (7.8–12.7) (*N* = 31)	6.7 (6.2–7.2) (*N* = 7)
Hemoglobin, minimum value (median, g/dL)	6.5 (5.8–7,5)	6.0 (5.9–6.2) (*N* = 8)
Creatinine at admission <1y (median, μmol/L)	35 (31–250) (*N* = 3)	86 (61–110) (*N* = 2)
Creatinine at admission ≥1y (median, μmol/L)	135 (61–275) (*N* = 25)	115 (110–132) (*N* = 5)
Creatinine, maximum value <1y (median, μmol/L)	231 (197–348) (*N* = 3)	97 (67–126) (*N* = 2)
Creatinine, maximum value ≥1y (median, μmol/L)	355 (200–465) (*N* = 35)	228 (124–307) (*N* = 6)
eGFR at admission <1y (median, ml/min/1,73 m^2^)	42.8 (23.0–49.1) (*N* = 3)	21.8 (12.4–31.2) (*N* = 2)
eGFR at admission ≥1y (median, ml/min/1,73 m^2^)	16.4 (11.0–58.5) (*N* = 25)	19.4 (18.5–28.0) (*N* = 5)
eGFR, minimum value <1y (median, ml/min/1,73 m^2^)	6.5 (4.9–7.8) (*N* = 3)	21.6 (12.1–31.0) (*N* = 2)
eGFR, minimum value ≥1y (median, ml/min/1,73 m^2^)	15.0 (6.3–13.8) (*N* = 35)	13.9 (7.6–21.8)
LD^a^ at admission (median, U/L)	2241 (1153–2728) (*N* = 17)	2075 (1863–2659) (*N* = 5)
LD, maximum value (median, U/L)	3146 (2559–4023)	3090 (2441–5931) (*N* = 7)
Platelet count at admission (median, ×10^9^/L)	59 (39–175) (*N* = 30)	39 (24–107) (*N* = 7)
Platelet count, minimum value (median, ×10^9^/L)	32 (20–50)	24 (19–55) (*N* = 8)
CRP^b^ at admission (median, mg/L)	14 (9–30) (*N* = 30)	13 (2–21) (*N* = 6)
CRP, maximum value (median, mg/L)	67 (19–138) (*N* = 37)	29 (15–161) (*N* = 7)
WBC^c^ count at admission (median, ×10^9^/L)	17.0 (11.2–25.4) (*N* = 29)	11.6 (9.4–14.1) (*N* = 7)
WBC count, maximum value (median, ×10^9^/L)	19.4 (15.1–29.4)	16.0 (14.4–17.4) (*N* = 8)
Sodium at admission (median, μmol/L)	134 (130–137) (*N* = 27)	135 (130–135) (*N* = 6)

Results are presented as number of cases, *n* (%) and median (interquartile range). If data on the feature was not available in all medical records, the number of cases where available is presented with (*N* = number of cases where available). Estimated glomerular filtration rate (eGFR) was estimated retrospectively using the height-independent Pottel eGFR equation [29]. *HUS* hemolytic uremic syndrome

^a^ *LD* Lactate dehydrogenase

^b^ *CRP* C-reactive protein

^c^ *WBC* white blood cell

Table 3 presents the laboratory values registered for the two groups. Notably, in the 31 patients in the D^+^HUS where available, median hemoglobin value at admission was 11.1 g/dL.

## Discussion

In this nationwide retrospective survey on clinical, therapeutic and long-term aspects of hemolytic-uremic syndrome (HUS) in children in Norway, we describe the multiorgan burden of this life threatening disease. A substantial amount of the patients had a complicated inhospital period and long term renal complications were common. Over a 10 year period, a total of 47 HUS cases were identified in the period [5]. D^+^HUS was most common with 38 (80 %) cases, of which 23 (61 %) had confirmed STEC infection. There were nine (19 %) D^−^HUS cases; two were caused by pneumococci, three were of genetic origin, one was specified as caused by campylobacter and the remaining three had unknown etiology. Because of the low number of cases and the diverse etiologies comprising the D^−^HUS group, direct comparison between these groups was difficult. Similar studies exist on the HUS in other countries; with this work we have presented data on the HUS situation in Norwegian children, on which knowledge has been limited.

All but one of the confirmed STEC cases presented with diarrhea. Two of the D^−^HUS cases initially presented with bloody diarrhea; these had documented atypical causes, and were classified as D^−^HUS. This reflects the fact that some atypical HUS cases may present with diarrhea and emphasizes the importance of thorough diagnostic work to avoid potentially misdiagnosed cases based on early clinical presentation [4, 13]. This underlines one of the advantages of a more specific classification of HUS. Concomitantly, the relatively low frequency of these occurrences in our study also indicates that the D^+^/D^−^ classification may be useful, especially in early etiological considerations. Another point related to initial presentation is that the median value of hemoglobin at admission was 11.1 g/dL in the 31 D^+^HUS cases where available. A high level of hemoglobin at admission and even at diagnosis has also been described elsewhere [24]. This may reflect serious dehydration or that some patients were admitted before the most acute phase of hemolysis. In either case, this may be misleading in the early diagnostic work; an important point to consider when approaching a case with the initial clinical presentation of STEC infection and D^+^HUS.

Our data on the D^+^HUS group were in accordance with other studies concerning urine production at the time of admission; 76 % were oligoanuric when admitted to hospital, and 58 % required dialysis [9, 30]. Neurological complications, including seizures, brain infarction and development of epilepsy, were seen in 24 % of the cases, which is comparable to other reports [16, 31, 32]. Other neurological complications were documented, such as brain oedema and neurocognitive problems. A recent publication showed impaired neuromotor outcome in all patients included [33]. Unfortunately, we have no comparable documentation on the long term neuromotor function in this study.

Interestingly, 61 % in the D^+^HUS group were treated with antibiotics prior to and/or during initial hospitalization, although for several indications. This included sepsis, which was documented in 29 %. There has been disagreement on the use of antibiotics in both STEC and STEC-HUS cases. The current consensus advices against the use of antibiotics in STEC infections because of an assumed increased risk for HUS development as a consequence of toxin release [34]. Studies have shown variable results, and the use of antibiotics depends on several factors requiring a more nuanced approach [35, 36]. The use of antibiotics in established STEC-HUS is more controversial, although studies have shown no influence on long-term outcome [34]. We were not able to examine potential effects of the use of antibiotics in our study as the time of administration was often unclear and the indication was variable.

In the D^+^HUS cases where data on follow-up was available 1 year or more following initial admission, 21 % had persistent hypertension, 32 % persistent proteinuria and 8 % developed chronic kidney disease, one with need of a kidney transplant. These numbers are comparable to those described in other studies [20, 37]. The results are likely overestimated as a consequence of selective patient follow-up according to disease severity. Interestingly, previous studies have shown that some patients can develop sequelae such as hypertension and proteinuria several years after initial admission, even when showing no signs of sequelae in early follow-up [37]. This had led to the recommendations of follow up controls for at least 5 years for D^+^HUS patients. The case fatality rate in the D^+^HUS group was 5 %. This rate varies between studies, and is often higher during outbreaks, but is usually considered 3–5 % [4, 21].

In the D^−^HUS group, five of the nine patients were oligoanuric and of these three needed dialysis. In the study from Constantinescu [11] the renal complications of the D^−^HUS group were seemingly more pronounced than we could document. However, the number of cases in our study was low, which could have influenced the results. It should also be noted that the cases included here were treated before Eculizumab was introduced as an effective treatment in genetic HUS [25].

Only two patients were diagnosed with SP-HUS. Both were severely sick in the acute phase as a complication of their pneumococcal infection. SP-HUS is generally considered more lethal in the acute phase and D^−^HUS associated with more frequent long-term complications than D^+^HUS [11, 22]. In our study, the long-term consequences documented in SP-HUS were sustained bilateral loss of hearing, epileptic activity and spastic hemiplegia, but none of the two patients died.

There were limitations in this study. Firstly, due to the small size of the groups and different etiologies of the D^−^HUS group, comparison between them and to other studies was difficult and only descriptive results are therefore presented. Secondly, there was one outbreak of STEC leading to HUS in the 10-year period described here. This occurred in 2006 and included nine patients with STEC-HUS caused by STEC O103:H25 [27]. This outbreak seemingly had an unusually high STEC-HUS to STEC ratio, potentially caused by a particularly virulent strain. The outbreak constitute one fourth of the D^+^HUS group in this study, and may have affected the results presented.

Another issue that has to be addressed is the inclusion criteria and the considerations around inclusion of some of the cases that did not strictly fulfill the criteria. Serum creatinine was chosen instead of the pRIFLE criteria because a pilot project revealed difficulties in retrospectively obtaining data on urinary output in the medical records. We did not predict the problem with such a steep rise from one to 2 years of age in these criteria. If followed categorically, this would have excluded five cases. Three D^+^HUS cases and one D^−^HUS case had clinical HUS and serum creatinine below 80 μmol/L, but above laboratory age-related reference level at the hospitals in question. We decided to include them as regular HUS cases. A second D^−^HUS case had two admissions with reduced kidney function and falling serum hemoglobin and platelets, albeit not below our criteria. This case had an extensive family history of genetic HUS, confirmed corresponding mutations and later had recurring milder episodes. This would be considered a partial HUS case, but we decided to include it, albeit not in all estimations of clinical aspects. A sixth patient died early in the acute phase, with s-hemoglobin value only documented at admission and higher than required in our criteria. This patient had confirmed STEC infection. These cases were included after consulting a clinician with expertise in pediatric nephrology.

These challenges highlight some important limitations to these types of studies, especially when evaluating an extensive amount of data. There are two important factors in particular that need to be commented. One; we designed the data collection form from a pilot study of HUS medical records to assess which parameters where both relevant and available. Two; data collected from medical records were subject to the standards of different hospitals, clinicians and the subjective (and objective) opinions of the latter. Certain parameters were generally fixed and difficult to misinterpret, others were not. For example; “duration of initial hospitalization” was not subject to misinterpretation as the dates followed the medical records. On the other hand, “time from first symptom to admittance” was subject to uncertainty according to how clinicians had perceived disease progression (e.g. “a couple of days” or similar).

Another limitation is the consistency in a retrospective survey of medical records to provide all necessary data. All medical records and associated charts were examined thoroughly. However, we were only able to obtain measures of blood pressure at admittance in 23 cases. This only allowed us to assess blood pressure at admittance in less than half the cases, illustrating the potential for missing data in retrospective surveys.

## Conclusions

We have presented the clinical features, therapeutic interventions and long-term aspects of hemolytic-uremic syndrome in children in Norway over a 10-year period. A nationwide collection of data has allowed us to include all cases that occurred within this time span, describing this life-threatening condition on which knowledge concerning disease burden and outcome was limited. The data reports on the multi-organ affection in this disease entity with a high numbers of serious complications. These include a considerable number of cases with severe complications from the central nervous system, with brain micro infarctions and edema and development of epilepsy, of cardiac nature, such as myocardial infarction, in the gastrointestinal tract, such as colonic perforation and subsequent peritonitis, the respiratory system, such as acute respiratory failure, and a large proportion developing sepsis in the acute phase. These data underline that HUS patients have to be monitored carefully for extra-renal involvement in the acute phase. There were also a considerable number of cases showing long-term kidney related sequelae. Children with symptoms suspicious of HUS should be treated at centers with experience and possibilities for thorough monitoring. Through this and previous studies, we would like to emphasize the importance of thorough long-term follow-up and the need for quality guidelines to ensure this aspect of patient care in patients affected by HUS.

## Abbreviations

aHUS, atypical HUS; AKI, acute kidney injury; CNS, central nervous system; D^+^HUS, diarrhea-associated HUS; D^−^HUS, non-diarrhea-associated HUS; eGFR, estimated glomerular filtration rate; ESRD, end-stage renal disease; HUS, hemolytic-uremic syndrome; LD, lactate dehydrogenase; SP-HUS, *Streptococcus pneumoniae*-associated HUS; STEC, Shiga toxin producing *Escherichia coli;* TMA, thrombotic microangiopathy
